# Detection of SARS-CoV-2 using qRT-PCR in saliva obtained from asymptomatic or mild COVID-19 patients, comparative analysis with matched nasopharyngeal samples

**DOI:** 10.1371/journal.pone.0252964

**Published:** 2021-06-10

**Authors:** Kenji Ota, Katsunori Yanagihara, Daisuke Sasaki, Norihito Kaku, Naoki Uno, Kei Sakamoto, Kosuke Kosai, Taiga Miyazaki, Hiroo Hasegawa, Ayumi Fujita, Masato Tashiro, Takeshi Tanaka, Koichi Izumikawa, Koya Ariyoshi, Hiroshi Mukae, Jiro Yasuda, Kouichi Morita, Shigeru Kohno

**Affiliations:** 1 Department of Laboratory Medicine, Nagasaki University Hospital, Nagasaki, Japan; 2 Department of Respiratory Medicine, Nagasaki University Hospital, Nagasaki, Japan; 3 Infection Control and Education Center, Nagasaki University Hospital, Nagasaki, Japan; 4 Department of Infectious Diseases, Nagasaki University Hospital, Nagasaki, Japan; 5 Institute of Tropical Medicine, Nagasaki University, Nagasaki, Japan; 6 Nagasaki University, Nagasaki, Japan; Waseda University: Waseda Daigaku, JAPAN

## Abstract

**Objectives:**

The accurate detection of severe acute respiratory syndrome–coronavirus 2 (SARS-CoV-2) is essential for the diagnosis of coronavirus disease 2019 (COVID-19). We compared the quantitative RT-PCR results between nasopharyngeal swabs and saliva specimens.

**Methods:**

A COVID-19 outbreak occurred on a cruise ship at Nagasaki port, Japan. We obtained 123 nasopharyngeal swabs and saliva each from asymptomatic or mild patients in the late phase of infection.

**Results:**

The intervals from the diagnosis to the sampling were 25.5 days for nasopharyngeal swabs and 28.9 days for saliva. The positive rate was 19.5% (24/123) for nasopharyngeal swabs and 38.2% (47/123) for saliva (*P* = 0.48). The quantified viral copies (mean ± SEM copies/5 μl) were 9.3±2.6 in nasopharyngeal swabs and 920±850 in saliva (*P* = 0.0006).

**Conclusions:**

The advantages of saliva specimens include positive rate improvement and accurate viral load detection. Saliva may be used as a reliable sample for SARS-CoV-2 detection.

## Introduction

The global outbreak of the coronavirus disease 2019 (COVID-19) caused by severe acute respiratory syndrome–coronavirus 2 (SARS-CoV-2) has been the biggest threat for the global community since its first report in late 2019 [1, 2]. Precise and widely available diagnostic testing is crucial in combating COVID-19. Furthermore, early viral detection from asymptomatic patients is crucial in reducing transmission [3]. To date, nucleic acid amplification assays using polymerase chain reaction (PCR) have been accepted as a standard method for detection of SARS-CoV-2. Nasopharyngeal swabs are generally the recommended specimen [4], though several problems exist especially during sample collection. First, it can lead to potential expansion of infection. A number of suspected patients are inevitably in contact with each other and healthcare workers at the test site. In order to avoid exposure, healthcare workers are required to wear personal protective equipment (PPE) [5, 6], of which, the broad distribution and availability is often a challenge especially before upcoming outbreaks. Second, it is invasive and causes discomfort to the tested person [7]. Consequently, a serial repeated test is not desirable to monitor the viral load. Third, the healthcare workers are required to receive training in order to ascertain the quality of specimen [8]. Though a well-trained healthcare worker may obtain a specimen from the desired nasopharynx in a limited amount time and difficult position, training and experience are required for every essential healthcare workers. For those reasons, simple, safe-to-obtain and reproducible specimens are needed.

To acquire specimens, saliva is spat into a bottle without discomfort, pain, or danger and with a minimum exposure to other transmittable patients. Further, the sampling does not require any technique or skills. In a previous study [9], saliva demonstrated high concordance with nasopharyngeal specimens for detecting respiratory viruses, including coronaviruses. In recent studies comparing saliva and nasopharyngeal specimen for the detection of SARS-CoV-2, saliva was reported to be more sensitive than nasopharyngeal specimens for patients with severe COVID-19 [10]. In addition, SARS-CoV-2 has consistently been detected in the saliva [11]. However, the comparison of viral detection efficiency between nasopharyngeal swabs and saliva in the late phase of asymptomatic to mild COVID-19 patients remains unclear.

In this study a number of people were diagnosed with COVID-19 on a large cruise ship. The ship arrived at Nagasaki port on January 29, 2020 for the purpose of supplying materials. On April 20, 2020 one person among the 623 crew members was diagnosed with COVID-19. Following this, the infection spread and a total of 144 patients were diagnosed with laboratory-confirmed infection of SARS-CoV-2 using nasopharyngeal swab specimens. Some of the patients with moderate to severe symptoms were transferred to the hospitals to receive advanced treatment, while others with asymptomatic or mild symptoms remained on the ship or were isolated in compartments. The health condition of every crew member was closely monitored, and a series of nasopharyngeal swab tests were conducted till negative results were obtained. In addition, saliva specimens were collected from the crew members at single arbitrary times during their stay on the ship.

We aimed to compare the positive rate and viral load of SARS-CoV-2 between nasopharyngeal swabs and saliva specimens of asymptomatic and mild COVID-19 patients and report the results.

## Methods

### Sample collection

A series of nasopharyngeal swab and saliva samples were obtained from COVID-19 patients who had been diagnosed by RT-PCR or loop-mediated isothermal amplification (LAMP). The days of the diagnosis were defined as day 1, and the durations from day 1 to each sample collection were recorded. Nasopharyngeal samples were obtained by a group of experienced physicians using sterile flocked swabs. Saliva samples were self-collected by spitting directly into a sterile tube, immediately after waking up, prior to eating or drinking. The samples were immediately transported to the laboratory department of Nagasaki university hospital and proceeded to nucleic acid amplification assays.

This study was approved by the Institutional Review Board of Nagasaki University Hospital (20061701). The consent was not obtained since the data were analyzed anonymously.

### SARS-CoV-2 detection

Nucleic acid amplification assays were conducted following manual published by the National Institute of Infectious Diseases (NIID) [12]. Total nucleic acids were extracted from nasopharyngeal swab samples using MagMAX-96 for Microarrays Total RNA Iolation Kit, and saliva samples using the MagMAX Viral/Pathogen Nucleic Acid Isolation kit (ThermoFisher Scientific) following the manufacturer’s protocol, respectively. 200 μL of saliva specimen and a whole nasopharyngeal swab were eluted into 100 μl of elution buffer, respectively. Time and effort required for sample preparation did not differ significantly between these two sample types. For SARS-CoV-2 RNA detection, 5 μl of RNA template was tested, using real-time reverse transcription (RT)-PCR primer (forward AAATTTTGGGGACCAGGAAC, reverse TGGCAGCTGTGTAGGTCAAC) /probe (5’-FAM- ATGTCGCGCATTGGCATGGA-BHQ1-3’) sets for 2019-nCoV_N2. PCR was conducted using Thunderbird probe one-step qRT-PCR Kit (TOYOBO) and a Rotor-Gene Q 5plex HRM System (QIAGEN). The PCR program consisted of 95°C for 5 min followed by 50 cycles of 95°C for 10 s and 60°C for 30 s. Viral copies were quantified using a standard curve of a 10-fold serial dilution of RNA transcripts provided by the NIID. Viral load was expressed as copies/5 μl.

### Statistical analysis

All data were analyzed using Prism ver. 7.0e (GraphPad Software). A *P* value of <0.05 was considered to indicate a statistically significant difference.

## Results

### Sampling information

**Fig 1:**
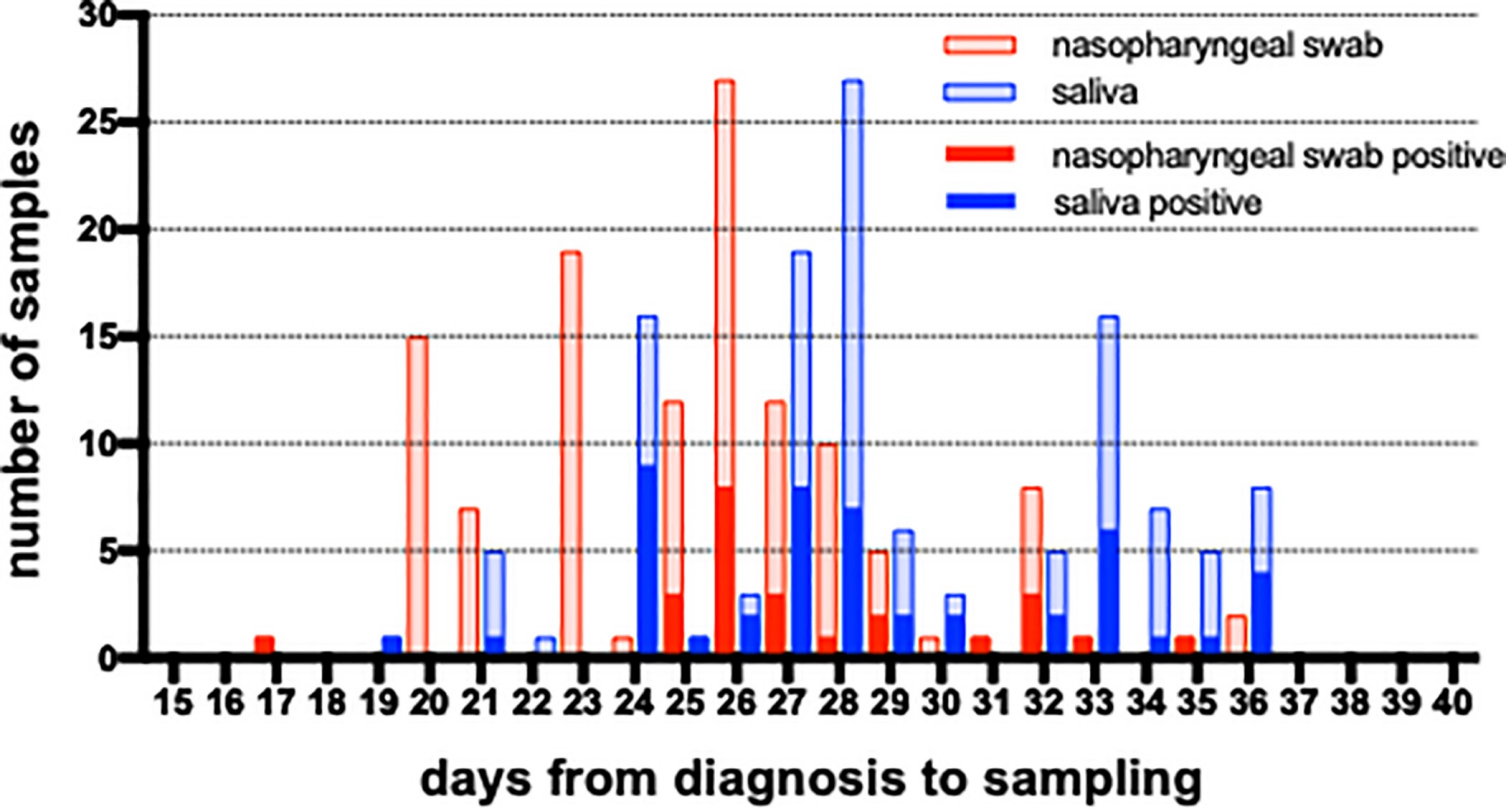
The numbers of matched nasopharyngeal and saliva samples. The numbers of tested samples are shown as light color and positive result as dark.

A total of 247 nasopharyngeal swab and 123 saliva specimens were obtained from 123 COVID-19 patients. The number of samples and timepoints are shown in S1 Fig. Among them, 123 pairs of nasopharyngeal swabs and saliva specimens each from 123 patients were matched (Fig 1) and included in the following analysis. The result of a nasopharyngeal swab sample obtained at the nearest date to that of the saliva sample was chosen to draw a comparison. The intervals from the diagnosis to the sampling were 25.5 days for nasopharyngeal swabs and 28.9 days for saliva. For 110 of the 123 cases (89.4%; nasopharyngeal swabs and saliva specimen pairs), nasopharyngeal swab samples were collected earlier than saliva specimens, with a median gap of 2 days.

### Quantitative result concordance between nasopharyngeal swab and saliva

The number of positive and negative samples are shown in Table 1. The overall concordance rate was 60.2% (74/123). In the group with positive result of nasopharyngeal swab, 45.8% (11/24) of saliva samples were positive, whereas only 23.4% (11/47) nasopharyngeal swab samples were positive among the group with positive result of saliva. The positive rate of each specimen was 19.5% (24/123) for nasopharyngeal swab and 38.2% (47/123) for saliva (*P* = 0.48, Fisher’s exact test).

**Table 1:** The number of SARS-CoV-2 RT-PCR results for nasopharyngeal swab and saliva.

		saliva		total
		+	‒	
nasopharyngeal swab	+	11	13	24
	‒	36	63	99
total		47	76	123

The positive/negative numbers of PCR result for each specimen are shown.

### Results of quantification (qRT-PCR)

**Fig 2:**
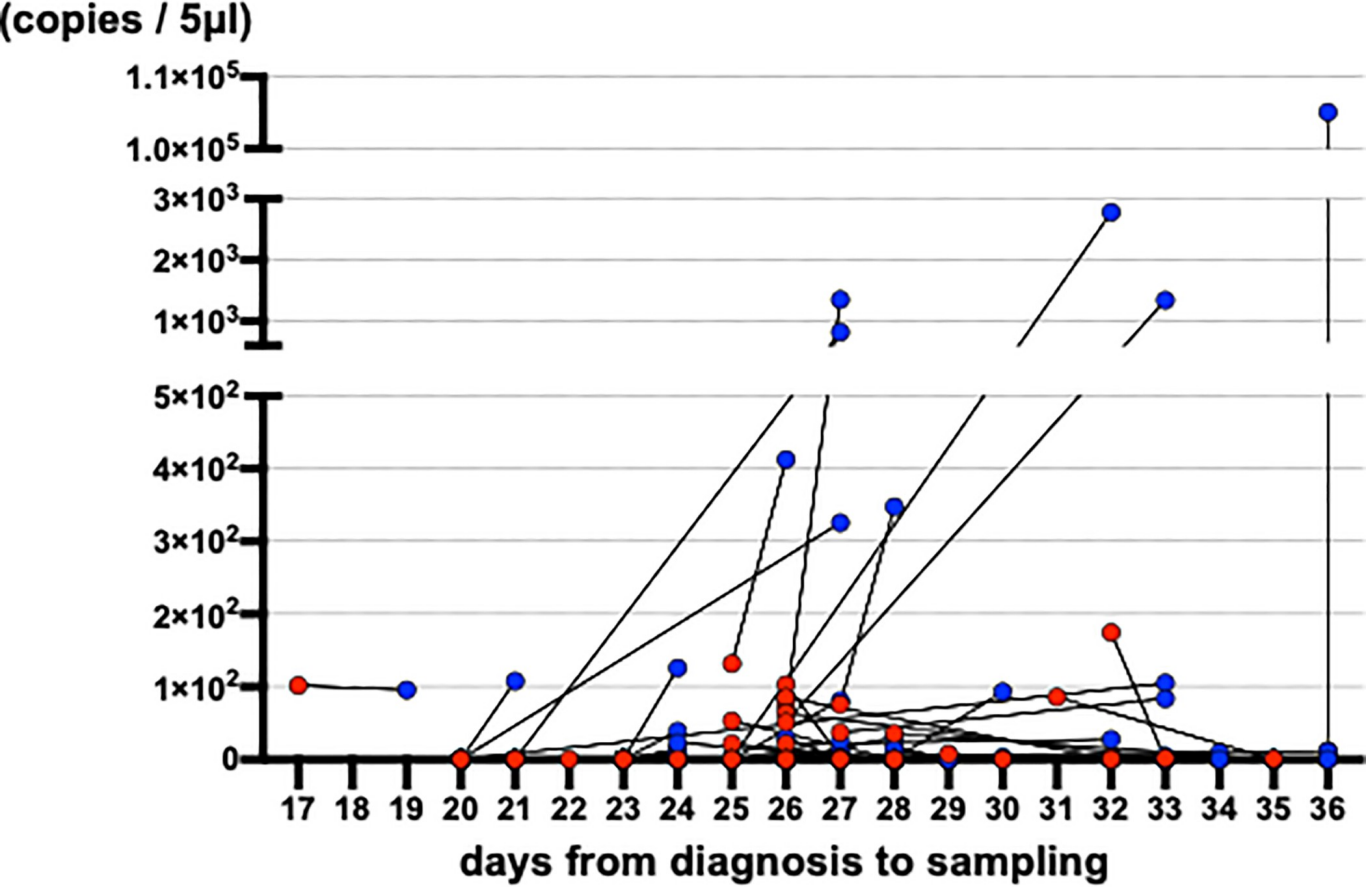
The quantitated virus copies of matched nasopharyngeal swab and saliva on timeline.

**Fig 3:**
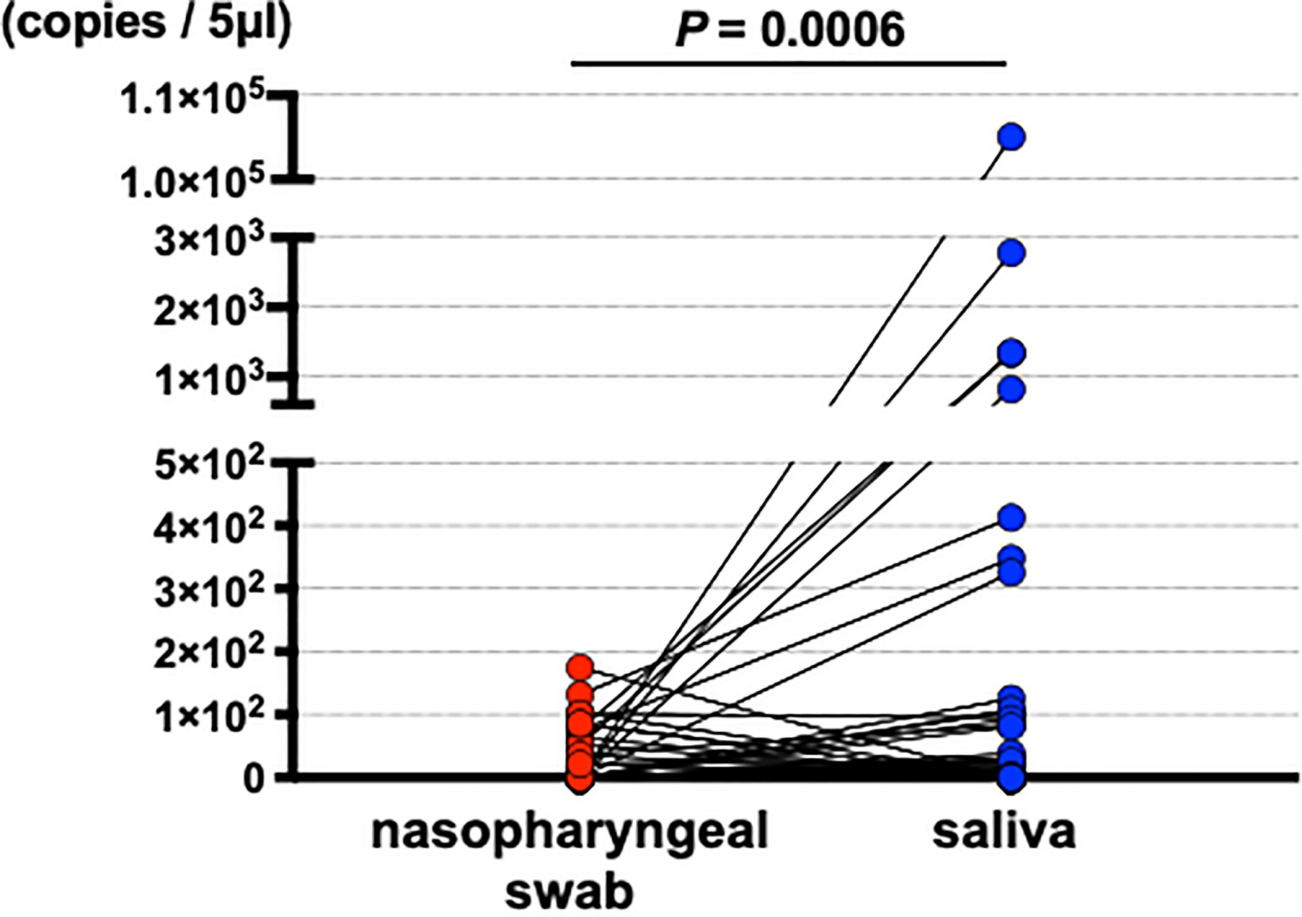
The virus copies of paired samples (n = 123) were compared and analyzed by Wilcoxon matched-pairs signed rank test.

**Fig 4:**
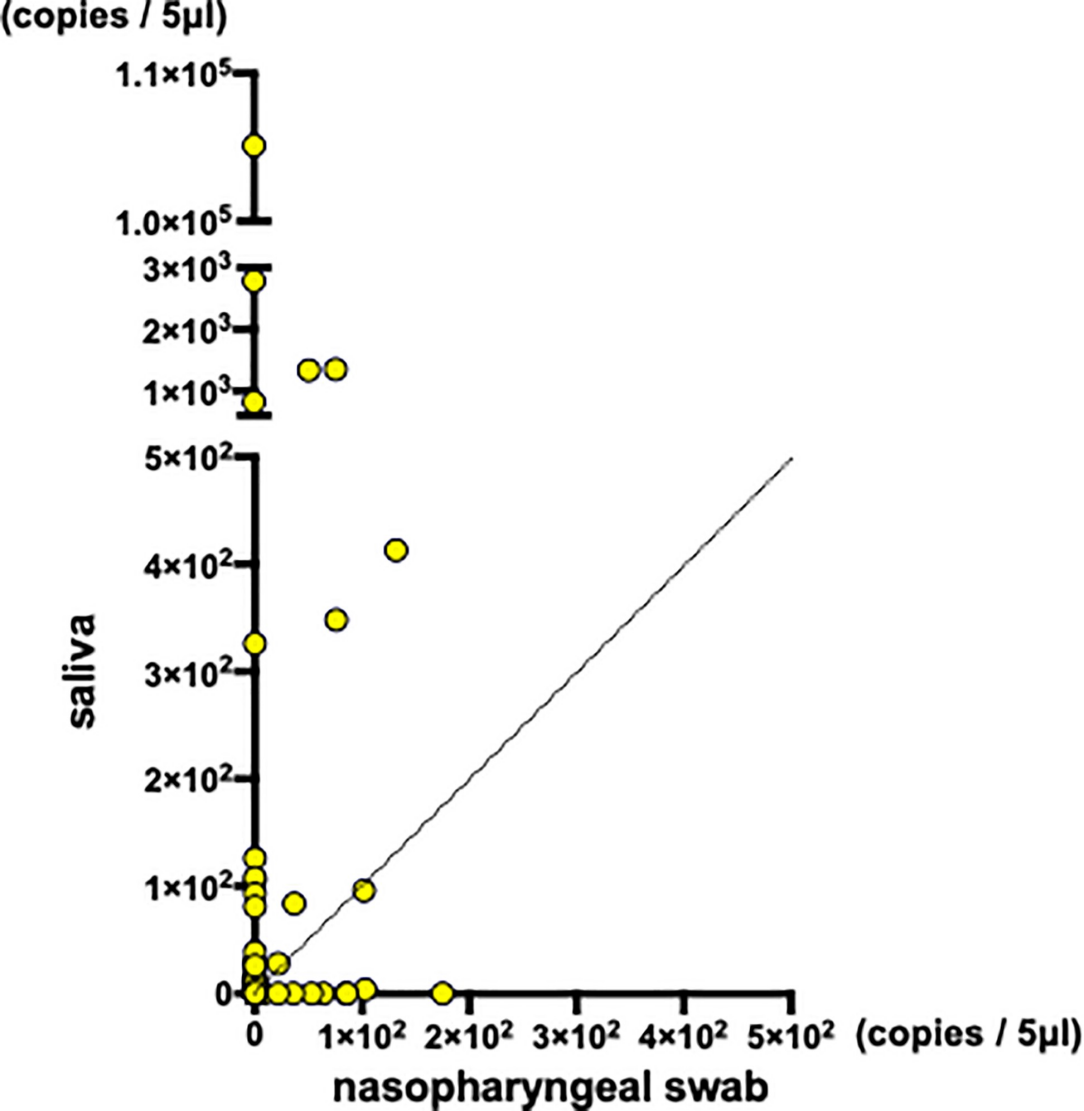
The paired quantitated virus copies of nasopharyngeal swab and saliva on scatter plot (n = 123).

Quantitated viral copies including all of the samples are shown in timeline in S2 Fig. Matched comparisons of quantitated viral copies on the timeline are shown in Fig 2. Matched comparisons of quantitated viral copies were analyzed. Increased viral copies were detected in saliva samples (Fig 3 and S3A, S3B Fig). With mean viral copies ± SEM of 9.3 ± 2.6 copies/5 μl detected in nasopharyngeal swabs and 9.2 × 10^2^ ± 8.5 × 10^2^ copies/5 μl detected in saliva specimens (*P* = 0.0006, Wilcoxon matched-pairs signed rank test). Among the 123 matched-pairs, increased viral copies were detected in saliva specimens for 44 pairs (35.8%), while increased viral copies were detected in nasopharyngeal swabs for 16 pairs (11.1%) (Fig 4). The viral copies of the rest 63 pairs (51.2%) were undetectable.

## Discussion

In this study the advantages of saliva specimens in detecting SARS-CoV-2 were demonstrated.

Wyllie et al. [10] reported increased sensitivity of the saliva specimens compared to that of the nasopharyngeal swab specimens for severe COVID-19 patients in early phase of 1 to 5 days, 6 to 10 days, and 11 or more days after the diagnosis. They also reported continuous viral detection in saliva specimens. Our results add important findings to saliva specimen’s high sensitivity even in late phase of asymptomatic to mild COVID-19 patients. In this pandemic, SARS-CoV-2 PCR test is performed not only for disease diagnosis but for incidence surveillance. In fact, the Japanese Government is conducting a surveillance to monitor COVID-19 incidence using saliva specimen. High sensitivity of saliva can be useful in detecting SARS-CoV-2 in surveillance situation. In addition, persistent shedding virus is helpful in obtaining sample for further examination, like detecting variant of concern / interest or whole genome sequencing for phylogenetic analysis. Recent systematic reviews indicate comparable sensitivity and specificity of saliva specimen compared to nasopharyngeal specimens [13, 14], supporting economic advantage and expansion of testing access. In our study, SARS-CoV-2 was detectable in 38.2% of saliva specimens and in 19.5% of the nasopharyngeal swabs. These results indicate that, overall, saliva specimens are more sensitive than nasopharyngeal swabs for the detection of SARS-CoV-2 from asymptomatic or mild COVID-19 patients. Some patients had viral loads below the detection limit of saliva samples, with a corresponding nasopharyngeal swab showing a positive result. However, among the 47 patients with positive saliva samples, only 23.4% (11/47) of saliva samples and nasopharyngeal swabs were positive simultaneously, which indicates an increased detection rate for saliva compared to that for nasopharyngeal swabs.

Increased viral copies were also demonstrated in saliva samples in a recent report [10]. Angiotensin-converting enzyme II (ACE2) is the cell surface receptor of the host for SARS-CoV-2 cell invasion [15]. ACE2 expression in salivary glands and on the tongue has been reported [16], which supports its role as an invasion cue. Since droplets generated by saliva are considered to be infectious [16], viral detection from saliva may support its infectivity. However, further studies are required to investigate whether these virus detected from the saliva samples are infectious [11].

Though saliva specimens are considered to be an infectious source, the virus may be contained, with less chance of transmission to healthcare workers. In addition, saliva sampling does not induce sneezing, which increases the risks of infection, as nasopharyngeal swabbing does.

There are several limitations in this study. First, the samples were not obtained at the same time. Due to the circumstances and the chaos of the outbreak on a closed ship, collection of each sample at the exact same time proved to be difficult. Therefore, there may be a possibility that the viral load reflects a different phase of infection. However, in most cases (110/123), the saliva samples were collected later than the nasopharyngeal swab samples. That is, the higher positive rate and virus loads were demonstrated in saliva samples obtained at the later timing, which are considered to contain decreasing viral load. Higher viral load detected from saliva samples obtained later than nasopharyngeal swab might reflect viral shedding. Though further studies are required to this speculation, statistically higher viral load was observed from saliva as clinical laboratory test in this study, which we believe to have a potential of contribution to more sensitive detection of COVID-19 in clinical setting. Moreover, our finding provides further information to clinicians that a few days gap in obtaining specimens is acceptable. In combatting COVID-19 cluster in similar cases such as our case, with limited medical staffs and resources, simultaneous sampling can sometimes be difficult. Our result enables clinicians to develop more flexible strategy in confused setting. Second, this study does not include moderate to severe COVID-19 patients. Since this study was conducted using specimens collected from the crew members of a cruise ship, where medical support is not adequately provided, patients who required advanced care could not be included. Further clinical studies are warranted to clarify the comparison between specimens based on the severities and phases of COVID-19. Third, the samples analyzed in this study were obtained after the first diagnosis of each patient. As the patients included in this study remained on the ship, these tests were conducted to re-affirm negative results. Nasopharyngeal specimens were thus obtained 25.5 days and saliva 28.9 days after the first diagnosis. Further studies are required to determine the sequential dynamics of the viral load from these specimens.

## Conclusion

In this study, we demonstrated that saliva specimens were superior to nasopharyngeal swab specimens for the detection of SARS-CoV-2 in the late phase of asymptomatic or mildly symptomatic COVID-19 patients. The saliva specimen may be used as a reliable option for the rapid viral detection of SARS-CoV-2.

## Supporting information

S1 Fig The numbers of total (prior to matching) nasopharyngeal and saliva samples. The numbers of tested samples are shown as light color and positive result as dark.
(TIFF)

S2 Fig The quantitated virus copies of total (prior to matching) nasopharyngeal swab and saliva on timeline. (TIFF)

S3 Fig The virus copies of paired samples (shown in S3 Fig) split into S3a (saliva > nasopharyngeal swab) and S3b (nasopharyngeal swab > saliva). (ZIP)

## Abbreviations

COVID-19: coronavirus disease 2019
SARS-CoV-2: severe acute respiratory syndrome–coronavirus 2
PCR: polymerase chain reaction
LAMP: loop-mediated isothermal amplification
PPE: personal protective equipment
NIID: National Institute of Infectious Diseases
RT: reverse transcription
SEM: standard error of the mean
ACE2: Angiotensin-converting enzyme II

## References

[1] Chan JF-W, Yuan S, Kok K-H, To KK-W, Chu H, Yang J, et al. A familial cluster of pneumonia associated with the 2019 novel coronavirus indicating person-to-person transmission: a study of a family cluster. Lancet [Internet]. 2020 Feb 11;395(10223):514–23. Available from: https://academic.oup.com/jid/article/221/11/1757/5739751 doi: 10.1016/S0140-6736(20)30154-9 31986261 PMC7159286

[2] Huang C, Wang Y, Li X, Ren L, Zhao J, Hu Y, et al. Clinical features of patients infected with 2019 novel coronavirus in Wuhan, China. Lancet. 2020;395(10223):497–506. doi: 10.1016/S0140-6736(20)30183-5 31986264 PMC7159299

[3] Anne Kimball; Kelly M. Hatfield; Melissa Arons; Allison James; Joanne Taylor; Kevin Spicer; et al. Asymptomatic and Presymptomatic SARS-CoV-2 Infections in Residents of a Long-Term Care Skilled Nursing Facility—. Morb Mortal Wkly Rep Summ CDC. 2020;69(13):377–81. 10.15585/mmwr.mm6913e1 PMC7119514 32240128

[4] Wölfel R, Corman VM, Guggemos W, Seilmaier M, Zange S, Müller MA, et al. Virological assessment of hospitalized patients with COVID-2019. Nature. 2020;(March). 10.1038/s41586-020-2196-x 32235945

[5] World Health Organization. Infection prevention and control during health care when novel coronavirus (nCoV) infection is suspected [Internet]. 2020. p. 1–5. Available from: https://www.who.int/publications/i/item/10665-331495

[6] National Center for Immunization and Respiratory Diseases, Division of Viral Diseases. Interim Infection Prevention and Control Recommendations for Patients with Suspected or Confirmed Coronavirus Disease 2019 (COVID-19) in Healthcare Settings. Cdc [Internet]. 2020;2:1–10. Available from: https://www.cdc.gov/coronavirus/2019-ncov/infection-control/control-recommendations.html

[7] Frazee BW, Rodríguez-Hoces de la Guardia A, Alter H, Chen CG, Fuentes EL, Holzer AK, et al. Accuracy and Discomfort of Different Types of Intranasal Specimen Collection Methods for Molecular Influenza Testing in Emergency Department Patients. Ann Emerg Med. 2018;71(4):509–517.e1. doi: 10.1016/j.annemergmed.2017.09.010 29174837

[8] Qian Y, Zeng T, Wang H, Xu M, Chen J, Hu N, et al. Safety management of nasopharyngeal specimen collection from suspected cases of coronavirus disease 2019. Int J Nurs Sci [Internet]. 2020 Apr;7(2):153–6. Available from: https://linkinghub.elsevier.com/retrieve/pii/S2352013220300521 doi: 10.1016/j.ijnss.2020.03.012 32292635 PMC7129371

[9] Y.-G. K, S.G. Y, M.Y. K, K. P, C.H. C, S.Y. Y, et al. Comparison between saliva and nasopharyngeal swab specimens for detection of respiratory viruses by multiplex reverse transcription-PCR. J Clin Microbiol [Internet]. 2017;55(1):226–33. Available from: http://www.embase.com/search/results?subaction=viewrecord&from=export&id=L613997056%0A 10.1128/JCM.01704-16 27807150 PMC5228234

[10] Wyllie AL, Fournier J, Casanovas-Massana A, Campbell M, Tokuyama M, Vijayakumar P, et al. Saliva or Nasopharyngeal Swab Specimens for Detection of SARS-CoV-2. N Engl J Med [Internet]. 2020 Sep 24;383(13):1283–6. Available from: http://www.nejm.org/doi/10.1056/NEJMc2016359 32857487 10.1056/NEJMc2016359 PMC7484747

[11] To KK-W, Tsang OT-Y, Yip CC-Y, Chan K-H, Wu T-C, Chan JM-C, et al. Consistent Detection of 2019 Novel Coronavirus in Saliva. Clin Infect Dis [Internet]. 2020 Feb 12;(Xx Xxxx):4–6. Available from: https://academic.oup.com/cid/advance-article/doi/10.1093/cid/ciaa149/5734265 10.1093/cid/ciaa149 PMC7108139 32047895

[12] National Institute of Infectious Diseases. Manual for the Detection of Pathogen 2019-nCoV Ver.2.6 [Internet]. 2020. p. 1–16. Available from: https://www.niid.go.jp/niid/images/epi/corona/2019-nCoVmanual20200217-en.pdf

[13] Bastos ML, Perlman-Arrow S, Menzies D, Campbell JR. The Sensitivity and Costs of Testing for SARS-CoV-2 Infection With Saliva Versus Nasopharyngeal Swabs. Ann Intern Med. 2021;1–12. doi: 10.7326/M20-0428 33428446 PMC7822569

[14] Butler-Laporte G, Lawandi A, Schiller I, Yao MC, Dendukuri N, McDonald EG, et al. Comparison of Saliva and Nasopharyngeal Swab Nucleic Acid Amplification Testing for Detection of SARS-CoV-2: A Systematic Review and Meta-analysis. JAMA Intern Med. 2020;1–8. 10.1001/jamainternmed.2020.8876 PMC7811189 33449069

[15] Zhou P, Yang X Lou, Wang XG, Hu B, Zhang L, Zhang W, et al. A pneumonia outbreak associated with a new coronavirus of probable bat origin. Nature. 2020;579(7798):270–3. doi: 10.1038/s41586-020-2012-7 32015507 PMC7095418

[16] Xu R, Cui B, Duan X, Zhang P, Zhou X, Yuan Q. Saliva: potential diagnostic value and transmission of 2019-nCoV. Int J Oral Sci. 2020;12(1). doi: 10.1038/s41368-020-0080-z 32300101 PMC7162686

