# Supplementary figures and images for: Detection of SARS-CoV-2 using qRT-PCR in saliva obtained from asymptomatic or mild COVID-19 patients, comparative analysis with matched nasopharyngeal samples
Source: PLoS One. 2021 Jun 10;16(6):e0252964. doi: 10.1371/journal.pone.0252964 (PMC8191987; doi:10.1371/journal.pone.0252964)

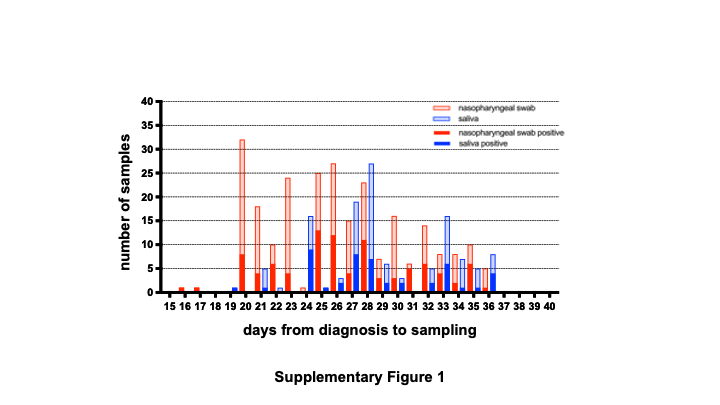

Supplement: S1 Fig — The numbers of tested samples are shown as light color and positive result as dark. (TIFF) [file pone.0252964.s001.tiff]

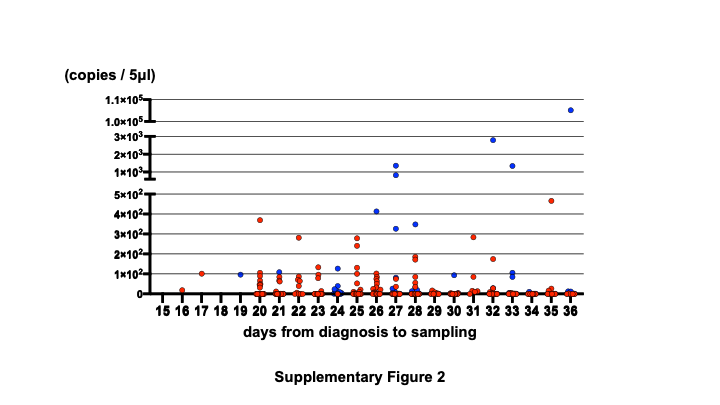

Supplement: S2 Fig — (TIFF) [file pone.0252964.s002.tiff]

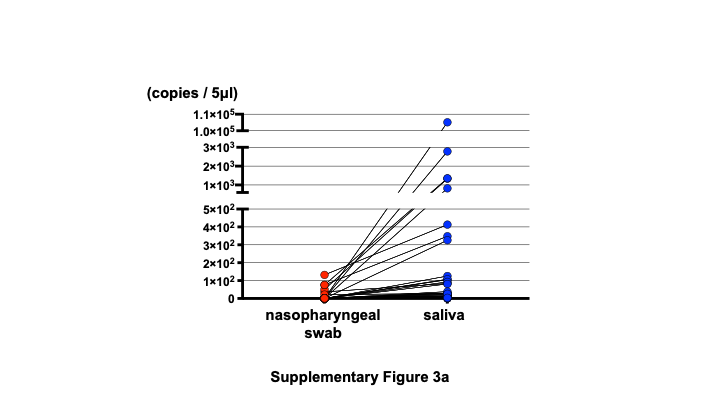

Supplement: S3 Fig — (ZIP) [file pone.0252964.s003.zip › S3a_Fig.tiff]

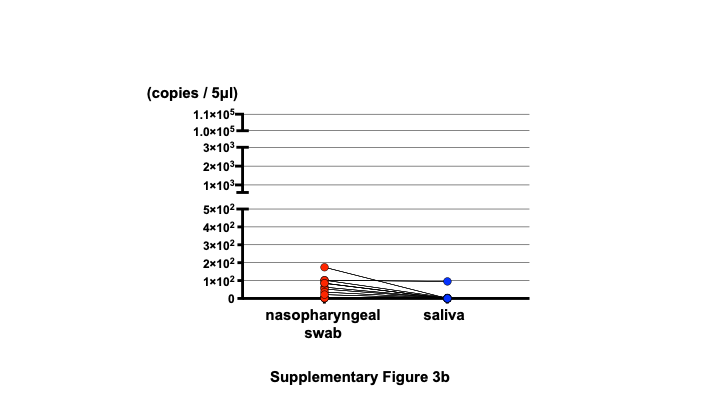

Supplement: S3 Fig — (ZIP) [file pone.0252964.s003.zip › S3b_Fig.tiff]
